# Coronary MR angiography at 3T: fat suppression versus water-fat separation

**DOI:** 10.1007/s10334-016-0550-7

**Published:** 2016-04-02

**Authors:** Maryam Nezafat, Markus Henningsson, David P. Ripley, Nathalie Dedieu, Gerald Greil, John P. Greenwood, Peter Börnert, Sven Plein, René M. Botnar

**Affiliations:** 1 Division of Imaging Sciences and Biomedical Engineering, King’s College London, London, UK; 2 Department of Medicine, Beth Israel Deaconess Medical Centre and Harvard Medical School, Boston, MA USA; 3 Multidisciplinary Cardiovascular Research Centre and the Division of Cardiovascular and Diabetes Research, Leeds Institute for Cardiovascular and Metabolic Medicine, University of Leeds, Leeds, UK; 4 Philips Research, Hamburg, Germany; 5 Pontificia Universidad Católica de Chile, Escuela de Ingeniería, Santiago, Chile

**Keywords:** Coronary magnetic resonance angiography, SPIR, Dixon, Vessel length, Vessel sharpness

## Abstract

**Objectives:**

To compare Dixon water-fat suppression with spectral pre-saturation with inversion recovery (SPIR) at 3T for coronary magnetic resonance angiography (MRA) and to demonstrate the feasibility of fat suppressed coronary MRA at 3T without administration of a contrast agent.

**Materials and methods:**

Coronary MRA with Dixon water-fat separation or with SPIR fat suppression was compared on a 3T scanner equipped with a 32-channel cardiac receiver coil. Eight healthy volunteers were examined. Contrast-to-noise ratio (CNR), signal-to-noise ratio (SNR), right coronary artery (RCA), and left anterior descending (LAD) coronary artery sharpness and length were measured and statistically compared. Two experienced cardiologists graded the visual image quality of reformatted Dixon and SPIR images (1: poor quality to 5: excellent quality).

**Results:**

Coronary MRA images in healthy volunteers showed improved contrast with the Dixon technique compared to SPIR (CNR _blood-fat_: Dixon = 14.9 ± 2.9 and SPIR = 13.9 ± 2.1; *p* = 0.08, CNR _blood-myocardium_: Dixon = 10.2 ± 2.7 and SPIR = 9.11 ± 2.6; *p* = 0.1). The Dixon method led to similar fat suppression (fat SNR with Dixon: 2.1 ± 0.5 vs. SPIR: 2.4 ± 1.2, *p* = 0.3), but resulted in significantly increased SNR of blood (blood SNR with Dixon: 19.9 ± 4.5 vs. SPIR: 15.5 ± 3.1, *p* < 0.05). This means the residual fat signal is slightly lower with the Dixon compared to the SIPR technique (although not significant), while the SNR of blood is significantly higher with the Dixon technique. Vessel sharpness of the RCA was similar for Dixon and SPIR (57 ± 7 % vs. 56 ± 9 %, *p* = 0.2), while the RCA visualized vessel length was increased compared to SPIR fat suppression (107 ± 21 vs. 101 ± 21 mm, *p* < 0.001). For the LAD, vessel sharpness (50 ± 13 % vs. 50 ± 7 %, *p* = 0.4) and vessel length (92 ± 46 vs. 90 ± 47 mm, *p* = 0.4) were similar with both techniques. Consequently, the Dixon technique resulted in an improved visual score of the coronary arteries in the water fat separated images of healthy subjects (RCA: 4.6 ± 0.5 vs. 4.1 ± 0.7, *p* = 0.01, LAD: 4.1 ± 0.7 vs. 3.5 ± 0.8, *p* = 0.007).

**Conclusions:**

Dixon water-fat separation can significantly improve coronary artery image quality without the use of a contrast agent at 3T.

## Introduction

Coronary artery magnetic resonance angiography (CMRA) requires effective fat suppression as coronary arteries are embedded in epicardial fat. Unwanted signal arising from fat can compromise vessel delineation and decrease the diagnostic value of CMRA [1].

Various fat suppression techniques have been proposed for CMRA such as short tau inversion recovery (STIR) and spectral presaturation with inversion recovery (SPIR) [2]. These methods are based on the relaxation time (T_1_) as well as chemical shift differences between fat and water. The frequency selective RF pulse of the SPIR technique only saturates the magnetization of fat while maintaining the magnetization of water and data acquisition begins immediately after the fat saturation pulse. In contrast, the STIR prepulse inverts both water and fat magnetization and imaging commences when the magnetization of fat is zero. However, each of these methods has specific drawbacks that may cause spatial misregisteration artifacts or sensitivity to *B*_0_ or *B*_1_ field inhomogeneities. Thus, it is important to develop and evaluate methods that may circumvent these drawbacks. STIR fat suppression suppresses the magnetization of fat and tissues which have similar short *T*_1_. As a result STIR is not recommended to be used in concert with a contrast agent. SPIR images have higher SNR compared to STIR images because the STIR prepulse inverts both the water and fat magnetization. For these reasons the SPIR technique has become the preferable fat suppression method for coronary artery imaging.

MRI chemical shift based water fat separation methods such as the Dixon method (first described by Dixon in 1984 [3]) provide excellent water and fat separation by acquiring images at carefully chosen echo times and using pixel by pixel image algebra. It provides additional diagnostic benefits (fat image) compared to conventional fat-saturation techniques [3–6]. The Dixon method has been shown to increase the image quality of whole heart CMRA at 1.5T [7]. Unlike other methods of water-fat separation that selectively excite water or suppress fat signal, the Dixon technique takes advantage of the phase shifts due to the water-fat resonance frequency difference in order to separate water from fat. In addition, the key advantage of the Dixon method is its tolerance to the main field inhomogeneity. This is achieved by including the underlying B0 distribution into the signal model avoiding adverse effects on the signal separation process this way. Furthermore, the separation of water and fat by generating water and fat only images may reduce the sensitivity to motion artifacts in the water images as those artifacts often arise from residual fat signal in the chest wall that subsequently may lead to ghosting artifacts if chest wall motion is not adequately corrected for.

The purpose of this study was to compare CMRA with Dixon water fat separation with SPIR fat suppression at 3T and to investigate the sensitivity of both methods to the larger field inhomogeneity at 3T without administration of a contrast agent.

## Materials and methods

This work was performed using a 3T MR scanner (Achieva, Philips Healthcare, Best, the Netherlands) equipped with a 32-element cardiac coil for signal detection. Data were acquired in eight healthy adult subjects (six male, two female, mean age 36 ± 11 years). All subjects provided written informed consent and study was approved by the institutional review board. Imaging parameters of the SPIR and Dixon CMRA sequence were identical in healthy subjects. Every subject received two different scans: (1) conventional whole heart CMRA with SPIR fat suppression and (2) whole heart CMRA with two-point Dixon water fat separation in the same imaging session.

### CMRA pulse sequences

**Fig. 1:**
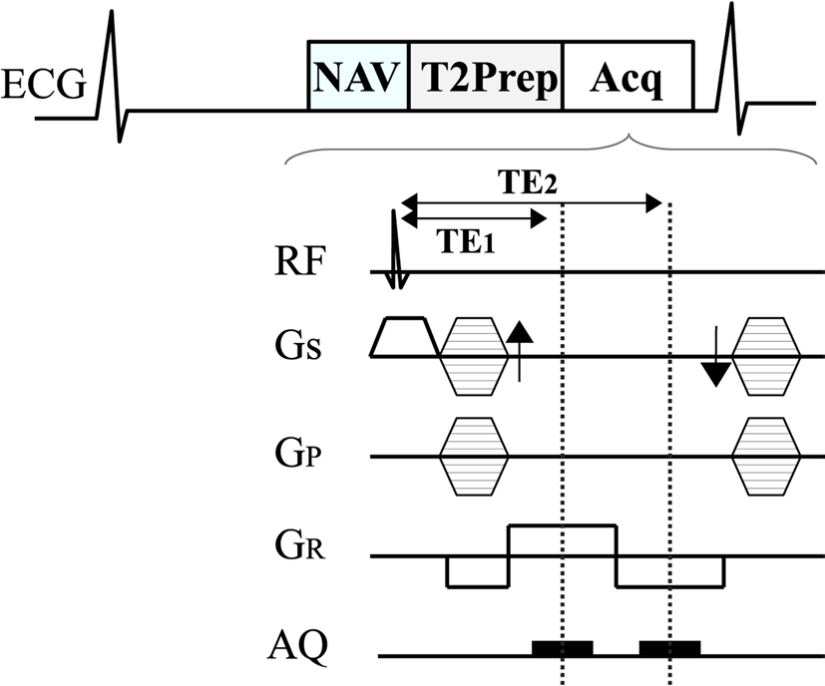
Schematic of ECG-triggered, magnetization prepared (T2-prepared) gradient echo imaging technique. *NAV* navigator echo for respiratory motion correction, *T2prep* T_2_ preparation prepulse for suppression of signal from myocardium and venous blood. *Acq* image acquisition, *TE* echo time

In this study, a segmented Turbo Field (TFE) gradient echo sequence was used both for the Dixon and SPIR CMRA. TFE was chosen because T_1_-weighted gradient echo imaging has been found to be superior to balanced gradient echo imaging at high field strength [8]. A schematic of the imaging sequence is shown in Fig. 1. The 3D CMRA sequence with isotropic spatial resolution was preceded by a pencil beam navigator for prospective respiratory motion correction [9, 10] and by a T_2_prep prepulse (TE_T2prep_ = 50 ms) for suppression of signal from myocardium and venous blood (with short *T*_2_ relaxation time) to improve contrast between arterial blood (*T*_2_ = 250 ms) and myocardium (*T*_2_ = 50 ms). Imaging was performed during the quiescent phase in mid-diastole and the gating window was 5 mm. Standard cardiac volume shim was used. Imaging parameters of the CMRA pulse sequence included: TR/TE = 4.1/1.7 ms for SPIR, TR/TE1/TE2 = 4.1/1.7/3.1 ms for Dixon, flip angle = 20°, acquisition window = 120 ms, slab thickness = 80–120 mm, FOV = 300 × 300 mm, matrix size = 448 × 448, in-plane resolution = 1.2 × 1.2 mm^2^, slice thickness = 1.2 mm, pixel bandwidth for SPIR = 1296 Hz/pixel and SENSE factor 2. Depending on the size of the heart, scans were performed either with 133 or 167 reconstructed slices. Images were acquired in the coronal plane with read-out in foot-head direction.

Imaging parameters were almost identical for the two-point Dixon and SPIR fat saturation. The readout gradient of the Dixon sequence consisted of two rephasing lobes with different polarity and echo times TE1 and TE2. In Dixon method the additional echo (TE1/TE2 = 1.7/3.1 ms) acquired did not alter the total scan time because the same repetition time (TR = 4.1 ms) was used for both methods to minimize parameter variations and to have comparable contrast and image quality. Bandwidth for the Dixon sequence was slightly higher to accommodate the two echoes and was 1568 Hz/pixel. Because of the two echoes and their actual timing, the SNR of the Dixon images is slightly higher (factor 1.2) than that of the SPIR images [11, 12]. Inline image reconstruction was performed to separate water and fat signal. Before separation of water and fat an appropriate phase correction is performed to eliminate phase errors induced by eddy currents [11].

### Image analysis

Two expert readers (15 and 3 years of cardiac MRI experience), blinded to the methods used, scored the image quality for each dataset using a five-point scale system: 1, poor quality; 2, structured visible but markedly blurred; 3, anatomy visible, but with moderate blurring; 4, minimal blurring; 5, well defined borders of vessel sharpness. All images were reformatted using dedicated software [13] to compare coronary artery delineation. Vessel sharpness and visualized vessel length of the RCA and LAD were quantified with Soapbubble software [13]. SNR was determined in fat $\left( {{\text{SNR}}_{\text{fat}} = \frac{{I_{\text{fat}} }}{{{\text{SDEV}}_{\text{fat}} }}} \right)$, blood and myocardium. Furthermore, CNR between blood, fat, $\left( {{\text{CNR}}_{\text{blood, fat}} = \frac{{I_{\text{blood}} { - }I_{\text{fat}} }}{{ 0. 5 ( {\text{SDEV}}_{\text{blood}} {\text{ + SDEV}}_{\text{fat}} )}}} \right)$ and myocardium were calculated.

### Statistical analysis

For statistical comparison a Wilcoxon Signed-Rank was used for the calculation of the image quality, whereas a *t* test was used for vessel sharpness and length. All measurements are presented as mean ±  standard deviation and *p* ≤ 0.05 considered statistically significant.

## Results

**Fig. 2:**
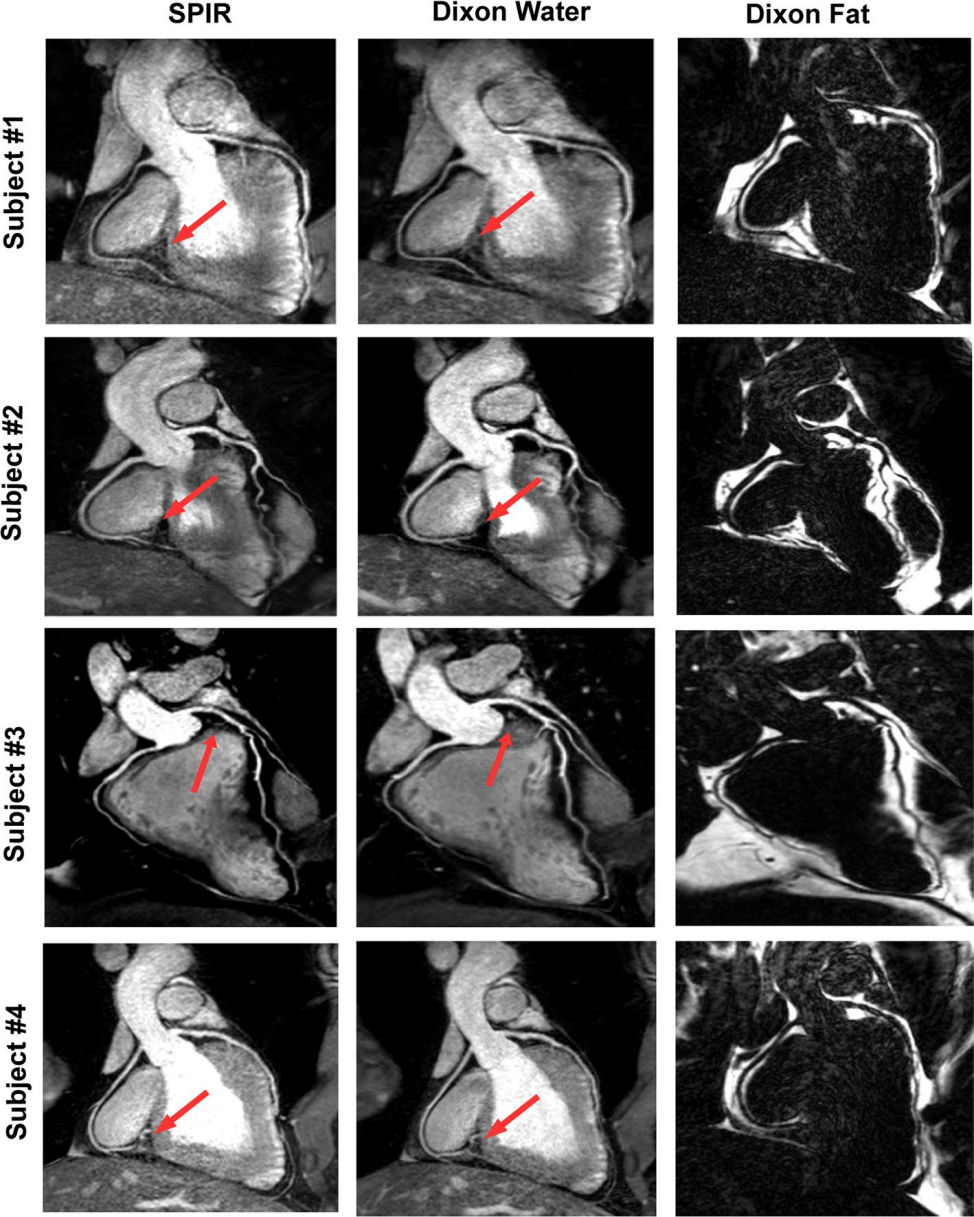
Reformatted whole heart CMRA using SPIR (*first column*) fat suppression and Dixon water (*second column*), fat (*third column*) separation in four representative volunteers. *Arrows* point to locations in the images where fat suppression was improved with the Dixon compared to the SPIR fat suppression method. Reformatted images demonstrate that fat suppression is good with both techniques at 3T without the use of a contrast agent. In addition, the Dixon technique provides a fat image which can be used for diagnostic purposes or to improve vessel tracking as distal segments are often displayed better in the fat images

All scans were successfully performed and produced good quality images in all volunteers. The total scan time for 133 reconstructed slices was Dixon: 365 ± 58 s and SPIR: 368 ± 58 s) and for 167 reconstructed slices (Dixon: 434.5 ± 15 s and SPIR: 445 ± 1 s). The average navigator efficiency was 54 ± 11 % (range 37–73 %). Figure 2 shows representative CMRA images from four healthy volunteers for both Dixon water-fat separation and SPIR fat suppression. Figure 2 demonstrates that excellent coronary artery image quality can be obtained at 3T without the use of a contrast agent. Fat suppression and vessel delineation with Dixon was visually superior to the SPIR technique (Fig. 2). Furthermore, images acquired in healthy subjects with the two-point Dixon method scored higher image quality than those acquired with SPIR (RCA: 4.6 ± 0.5 vs. 4.1 ± 0.7, *p* = 0.01, LAD: 4.1 ± 0.7 vs. 3.5 ± 0.8, *p* = 0.007).

Measured vessel length and sharpness of the RCA in healthy subjects acquired with both fat suppression methods are shown in Table 1. Vessel sharpness of the RCA acquired with the Dixon method was similar to SPIR fat suppression (57 ± 7 vs. 56 ± 9 %, *p* = 0.2). However, there were statistically significant differences found in the visualized vessel length for the Dixon compared to the SPIR fat suppression method (107 ± 21 vs. 101 ± 21 mm, *p* < 0.001). For LAD vessel sharpness (50 ± 13 vs. 50 ± 7 %, *p* = 0.4) and vessel length (92 ± 46 vs. 90 ± 47 mm, *p* = 0.4) both techniques performed equally well.

**Table 1:** Measured SNR, CNR, vessel sharpness, and length values and image quality scores with Dixon and SPIR sequence

	SNR fat	SNR blood	SNR myocardium	CNR blood and fat	CNR blood and myocardium	RCA vessel length (mm)	LAD vessel length (mm)	RCA vessel sharpness (%)	LAD vessel sharpness (%)	RCA score	LAD score
Dixon	2.1 ± 0.5	19.9 ± 4.5	10.4 ± 3.5	14.9 ± 2.9	10.2 ± 2.7	107 ± 21	92 ± 46	57 ± 7	50 ± 13	4.6 ± 0.5	4.1 ± 0.7
SPIR	2.4 ± 1.2	15.5 ± 3.1	8.2 ± 1.2	13.9 ± 2.1	9.11 ± 2.6	101 ± 21	90 ± 47	56 ± 9	50 ± 7	4.1 ± 0.7	3.5 ± 0.8
*p*-value	0.35	0.04	0.09	0.08	0.12	<0.001	0.4	0.2	0.4	0.01	0.007

The Dixon method was found to lead to similar fat suppression but increased SNR of blood and myocardium compared to SPIR fat suppression (Table 1). There was a trend towards higher CNR between blood and fat using the Dixon method (*p* = 0.08).

## Discussion

In this study, the two-point Dixon water-fat separation technique was compared with the SPIR fat suppression technique at 3T for non-contrast enhanced whole heart CMRA. The main findings of this study were that (1) there was improved image quality in terms of visual score, (2) there was a tendency for higher blood and myocardium SNR and blood/fat CNR, (3) longer visual vessel length and. Our findings are in agreement with a previous study at 1.5T where the Dixon water-fat separation technique helped to improve image quality of segmented TFE coronary MRA compared with SPIR fat suppressed balanced fast field echo (BTFE) CMRA [7].

Another advantage of the dual echo Dixon water fat separation technique compared to magnetization-prepared fat separation methods is that ghosting artifacts from not suppressed, moving, high intensity, chest wall fat only appears in the fat image and does not leak into the water image thereby minimizing sensitivity to breathing artifacts in the water image [7]. Because of the insensitivity of the chemical shift encoding to *B*_0_ and *B*_1_ inhomogeneities the robustness of Dixon fat suppression is better than that of SPIR at 3T. It has been shown that changing the direction of phase encoding in dual echo Dixon scans can reduce the ghosting level, increase the quality of the fat image and also reduce the motion sensitivity of the scan. Changing the phase encoding direction can increase scan time but if one is only interested in water images, image quality should be less dependent on the phase encoding direction [7]. Another advantage of Dixon-type fat-suppression strategy is its compatibility with steady-state acquisition, e.g. in cine-type acquisitions or recently proposed 4D coronary MRA [14, 15].

In the presence of B_1_ inhomogeneities (RF inhomogeneity), as typically encountered at 3T and higher field strengths, Dixon water fat separation should be superior to SPIR fat suppression because it is based on chemical shift difference and does not depend on the performance of saturation or inversion prepulses, which can be compromised by the transmit field inhomogeneity (*B*_1_^+^). In line with this, the overall performance of fat suppression was better for Dixon water-fat separation than for SPIR fat suppression in the current study.

We chose a wider receiver bandwidth for the Dixon compared to the SPIR to reduce chemical shifts artifacts and to allow faster data acquisition within the given sequence TR. Larger receiver bandwidth usually reduces SNR because more noise is included (SNR = 1/√rBW and chemical shift = 1/rBW), but this is compensated for by the dual echo acquisition and the noise averaging effect taking place in the Dixon reconstruction [11]. Moreover, due to its excitation bandwidth, the SPIR pulse may also saturate parts of the water peak, thereby reducing the SNR from the free water pool.

Although our data show no statistically significant differences between Dixon and SPIR in terms of coronary vessel sharpness, the two-point Dixon method improved the visual score and the visualized RCA length significantly.

Recent studies have demonstrated that cardiac fat may carry significant diagnostic value with a potential predictive value [7], such as better characterization of cardiac masses for identification of tumors, lipoma, and edema [16]. Visceral fat and obesity can cause different cardiovascular diseases such as cerebral vascular disease, coronary artery disease, and stroke [17–21]. Fibrofatty infiltrations of the myocardium have been found to be associated with a higher likelihood of sudden cardiac death [22] and arrythmogenic right ventricular cardiomyopathy (ARVC) is characterized pathologically by fibrofatty infiltration [23–26]. In addition, adipose tissue has been observed within the area of healed myocardial infarcts, with greater degree of fat volume in patients with coronary artery bypass graft surgery [27]. Moreover, pericardial fat volume is highly associated with atrial fibrillation (AF). For the above reasons, assessment of fatty infiltration by magnetic resonance imaging (MRI) may provide important diagnostic information for the detection and characterization of cardiovascular disease.

The additional fat data that is available with Dixon protocols may represent an important, until now clinically underused biomarker, and can be used to quantify the ratio of peri- to paracardial fat, and thus may enhance the diagnostic value of coronary MRA. In addition, as it is sometimes easier to find the tissue boundaries and the location of small epicardial vessels in the fat image rather than water image, the fat image also could be used to guide coronary artery segmentation. As a result the fat information can potentially be used for vessel tracking and characterization [7].

Contrast enhanced MR angiography (CE-MRA) techniques are widely used due to improved contrast-to-noise ratio (CNR) between blood and surrounding tissues, shorter examination times due to the ability to apply more imaging pulses because of shorter T_1_, better anatomical coverage and less flow artifacts compared to non-contrast MR angiography (NC-MRA). However, in high risk patients like patients with renal dysfunction or failure, NC-MRA is widely used. Moreover, NC-MRA does not require the perfect timing of the first pass of the contrast agent which can be challenging in the presence of cardiac or vascular disease. Furthermore contrast enhanced techniques are “single shot”, i.e., cannot be easily repeated, whereas NC-MRA allows multiple acquisitions if needed.

## Limitations

We only investigated a small number of healthy subjects in this proof-of-concept study. Further evaluation is needed to assess the diagnostic accuracy of the Dixon technique in patient population undergoing invasive clinical coronary angiography. In addition, the Dixon technique can be combined with an inversion recovery pulse for using in contrast enhanced coronary. However, further study is needed to compare Dixon and SPIR fat suppression in the presence of a contrast agent as fat and blood signal may have similar T_1_ , and thus fat suppression becomes important to delineate efficiently epicardial fat from coronary blood.

## Conclusions

We have demonstrated that the Dixon water-fat separation method provides improved CMRA image quality at 3T compared to the SPIR technique without the need of a contrast agent and may provide additional diagnostic information due to pericardial fat signal visualization without increasing the overall scan time.
